# Psychopathological characterization of nomophobia in a sample of patients with severe mental illness

**DOI:** 10.1192/j.eurpsy.2024.770

**Published:** 2024-08-27

**Authors:** G. Longo, R. Volgare, L. Orsolini, U. Volpe

**Affiliations:** ^1^Dipartimento di Neuroscienze Cliniche/DIMSC, Università Politecnica delle Marche, Ancona, Italy

## Abstract

**Introduction:**

Nomophobia, a neologism derived from the combination of “no mobile,” “phone” and “phobia,” represents one of the syndromes of today’s digital and virtual society. By this term, we refer to the discomfort, anxiety, nervousness, and distress generated by the individual’s loss of connection to his or her cell phone or other technological medium that allows connection to the Internet. No study has attempted to evaluate the impact of disconnection syndrome on a clinical sample of patients with Severe Mental Illness (SMI).

**Objectives:**

Our study has the objective of characterizing subject affected by SMI with nomophobia.

**Methods:**

Our study is conducted on inpatients (>16 years) referred to our Psychiatric ward in Ancona (Università Politecnica delle Marche, Italy). The following rating scales were administered to these subjects: Nomophobia Questionnaire (NMP-Q), Smartphone Addiction Scale - Short Version (SAS-SV), Multidimendional State Boredom Scale (MSBS), Intolerance of Uncertainty Scale (IUS), Temperament Evaluation in Memphis, Pisa and San Diego (TEMPS-M), Coping Orientation to the Problems Experiences-new Italian version (COPE-NVI).

**Results:**

Most of the subjects included in the study tested positive for nomophobia (99%; n=97). The mean score scored on the NMPQ is 69.2±27.9, while the mean score obtained at SAS-SV is 25.1±12.7. Gender has no influence on the scores obtained at the NMPQ (p=0.823), as well as the type of SMI (p=0.376). Those not in a relationship scored a higher mean score than who has a relationship (p=0.02). Patients who suffer from insomnia scored higher mean score on the NMPQ (p=0.21). A linear univariate regression between SAS-SV and NMPQ was observed (R^2^=0.575, F=129.731, p<0.001). A multivariate linear regression was observed between the NMPQ (R=0.556, R2=0.2830, F=12.057, p<0.001) and the IUS (B=1.343, p<0.001), the irritable temperament subscale of the TEMPS (B=1.293, p=0.003) and the inattention subscale of the MSBS (B=-1.029, p=0.033). In the men-only sample, a multivariate linear regression was observed between the NMPQ (R^2^=0.437, F=9.847, p<0.001) and the IUS (B=1.361, p<0.001), the anxious temperament subscale of the TEMPS (B=1.687, p=0.005) and the inattention subscale of the MSBS (B=-1.465, p=0.002).

**Conclusions:**

Patients with higher intolerance to uncertainty, irritable temperament and lower inattention have higher risk to develop nomophobia. In men with SMI, nomophobia is associated with higher intolerance to uncertainty, anxious temperament, and lower inattention. Further study have to be conducted to expand data and results.

**Disclosure of Interest:**

None Declared

